# Supplementary material for: Therapeutic Potential of a Novel αvβ3 Antagonist to Hamper the Aggressiveness of Mesenchymal Triple Negative Breast Cancer Sub-Type
Source: Cancers (Basel). 2019 Jan 24;11(2):139. doi: 10.3390/cancers11020139 (PMC6406933; doi:10.3390/cancers11020139)
Supplement: Supplementary file 1 [file cancers-11-00139-s001.pdf]

Supplementary Materials: Therapeutic potential of a novel $\alpha_v\beta_3$ antagonist to hamper the aggressiveness of mesenchymal triple negative breast cancer subtype

Billy Samuel Hill, Annachiara Sarnella, Domenica Capasso, Daniela Comegna, Annarita Del Gatto, Matteo Gramanzini, Sandra Albanese, Michele Saviano, Laura Zaccaro and Antonella Zannetti

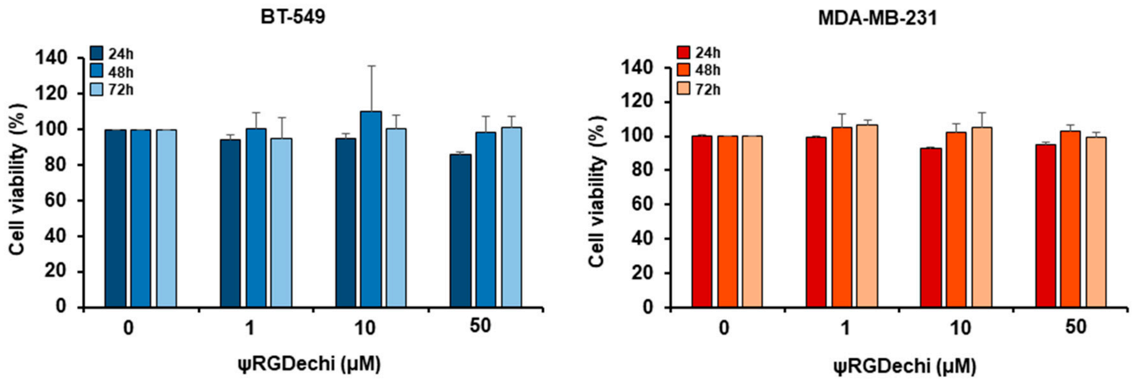

Figure S1. Effect of ψRGDechi on MES-TNBC cell proliferation. Viability of MDA-MB-231 and BT-549 cells (5.0 × 10^3 cells/well) untreated and treated with ψRGDechi (1 to 50 μM) at times 24, 48 and 72 hours by MTS Assay. Absorbance was read in a plate reader at a wavelength of 490 nm. The data are expressed as percentage of viable cells, considering the untreated control cells as 100%. Bars depict mean ± SD of three independent experiments.
